# Identifying the secondary electron cutoff in ultraviolet photoemission spectra for work function measurements of non-ideal surfaces

**DOI:** 10.1038/s41598-023-40187-5

**Published:** 2023-08-18

**Authors:** Arthur P. Baddorf

**Affiliations:** Center for Nanophase Materials Sciences, Oak Ridge National Laboratory, Oak Ridge, TN USA

**Keywords:** Condensed-matter physics, Nanoscale materials, Techniques and instrumentation, Characterization and analytical techniques

## Abstract

Absolute values of work functions can be determined in ultraviolet photoemission spectroscopy (UPS) by measuring the minimum kinetic energy of secondary electrons generated by a known photon energy. However, some samples can produce spectra that are difficult to interpret due to additional intensity below the true secondary electron cutoff. Disordered absorbates on elemental metals add small intensity below the onset for the transition metal surfaces studied, which can be attributed to energy losses after photoelectrons are generated. In contrast, spectra from WO_3−x_ films can produce multiple onsets with comparable intensity which do not fit this model. False onsets (in the context of work function measurements) can be minimized by optimizing experimental detection parameters including limiting analyzer acceptance angles and pass energy. True work functions can be identified by examining the onsets as the sample bias is varied.

## Introduction

The work function is defined as the minimum energy required to remove an electron from a material to vacuum. In addition to being a fundamental property related to the Fermi energy, it has practical implications for electron emission, contact potentials, chemical reactivity, and catalysis. Consequently, a number of approaches have been developed to determine a materials work function based on: electron emission (field emission, thermal emission, target current spectroscopy, scanning tunneling spectroscopy), contact potential (Kelvin probe, atomic force microscopy, diode current), and reactivity (ionization desorption)^1^. One of the most straightforward methods of measuring the absolute work function employs ultra-violet photoelectron spectroscopy (UPS) to generate electron emission^2^. With the sample grounded with respect to the electron detector, emission from the Fermi energy appears at a kinetic energy equal to the photon energy. The work function (ϕ) is then related directly to the photon energy (ℏω) and minimum kinetic energy of emitted electrons (E_k_) by:

1 $$ \phi \, = \,\hbar \omega \,{-}\,{\text{E}}_{{\text{k}}} . $$

This relationship was first established by Einstein in 1905^3^; Eq. (1) uses modern notation.

**Figure 1:**
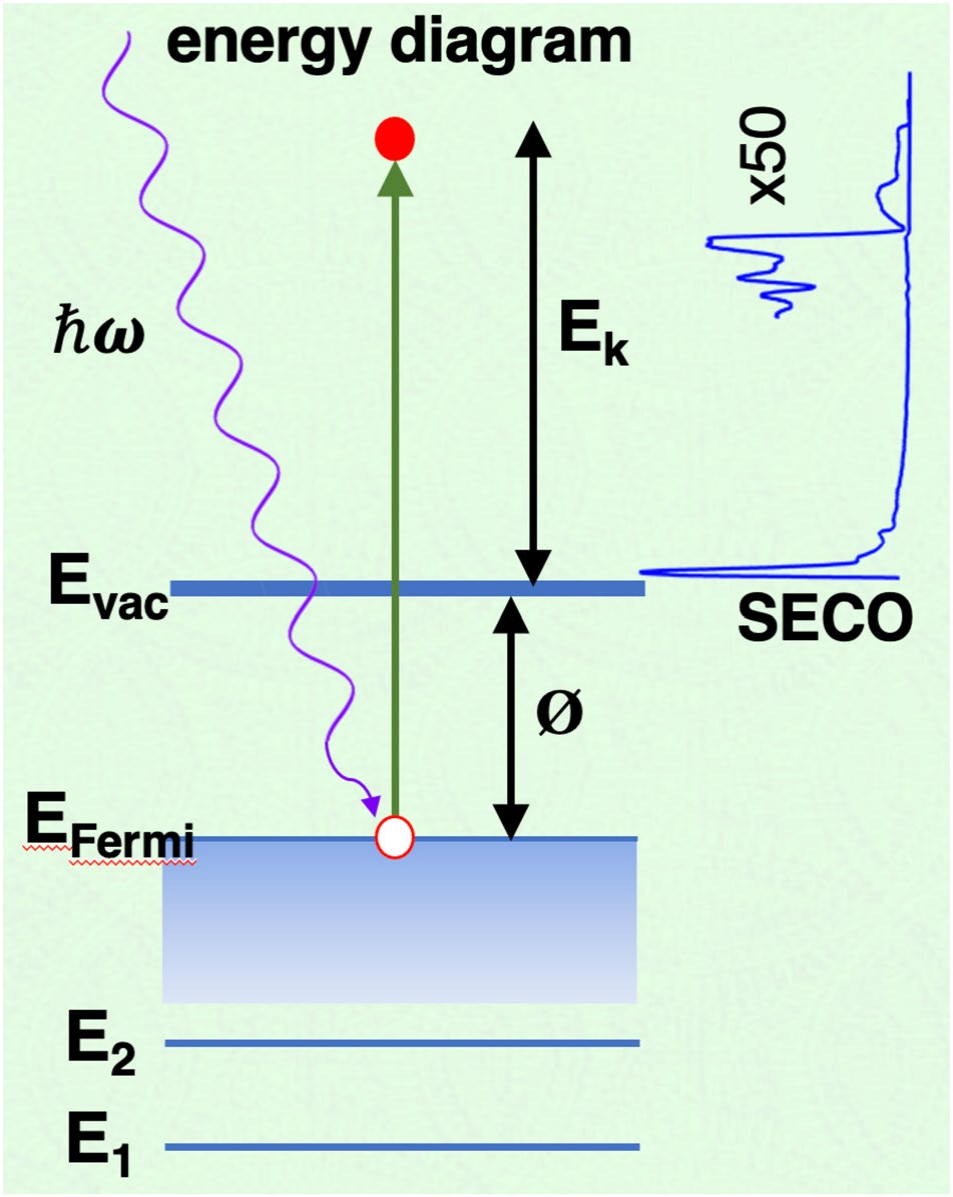
Energy schematic showing the relationship between photon energy (ℏω), work function (ϕ), and electron kinetic energy (*E*_*k*_). On the right is a typical UPS spectrum relating the secondary electron cutoff (at the bottom) to ϕ.

Figure 1 provides an energy diagram illustrating the relationship between ϕ, ℏω and E_k_. On the right is a typical UPS spectra showing that the entire energy width corresponds to ℏω − ϕ, or equivalently if the Fermi energy is assigned the value of ℏω then ϕ is the minimum observed kinetic energy. This minimum is commonly referred to as the secondary electron cut-off (SECO) as the low energy component is dominated by secondary electrons which typically provide a sharp cutoff in intensity.

While the relationship between work function and photoelectron spectra is straightforward theoretically, in practice complexities can arise. For example, electron analyzers and lens systems typically employed for UPS, e.g. hemispherical analyzers, do not work well at very low kinetic energies. As a result, in UPS measurements of work function the sample is biased negatively. This shifts the emission to higher kinetic energies where the minimum is more easily measured. However, both extrinsic fields from applied bias and intrinsic fields from the surface potential will distort electron trajectories. Consequently, Helander et al.^4^ have shown that the experimental geometry is important for accurate work function measurements with UPS, ideally with axial symmetry in the sample, mount, and lenses. In practice, orienting the surface normal toward the electron analyzer typically provides the highest available, although imperfect, symmetry.

This article discusses additional cases where the electron onset in UPS spectra is not ideal, clouding identification of the work function. These cases occur in more complex samples where a combination of factors may obscure the relationship between work function and photoemission experiment. Root causes for this complexity are explored and pragmatic solutions to interpret non-ideal spectra are offered.

## Results

**Figure 2:**
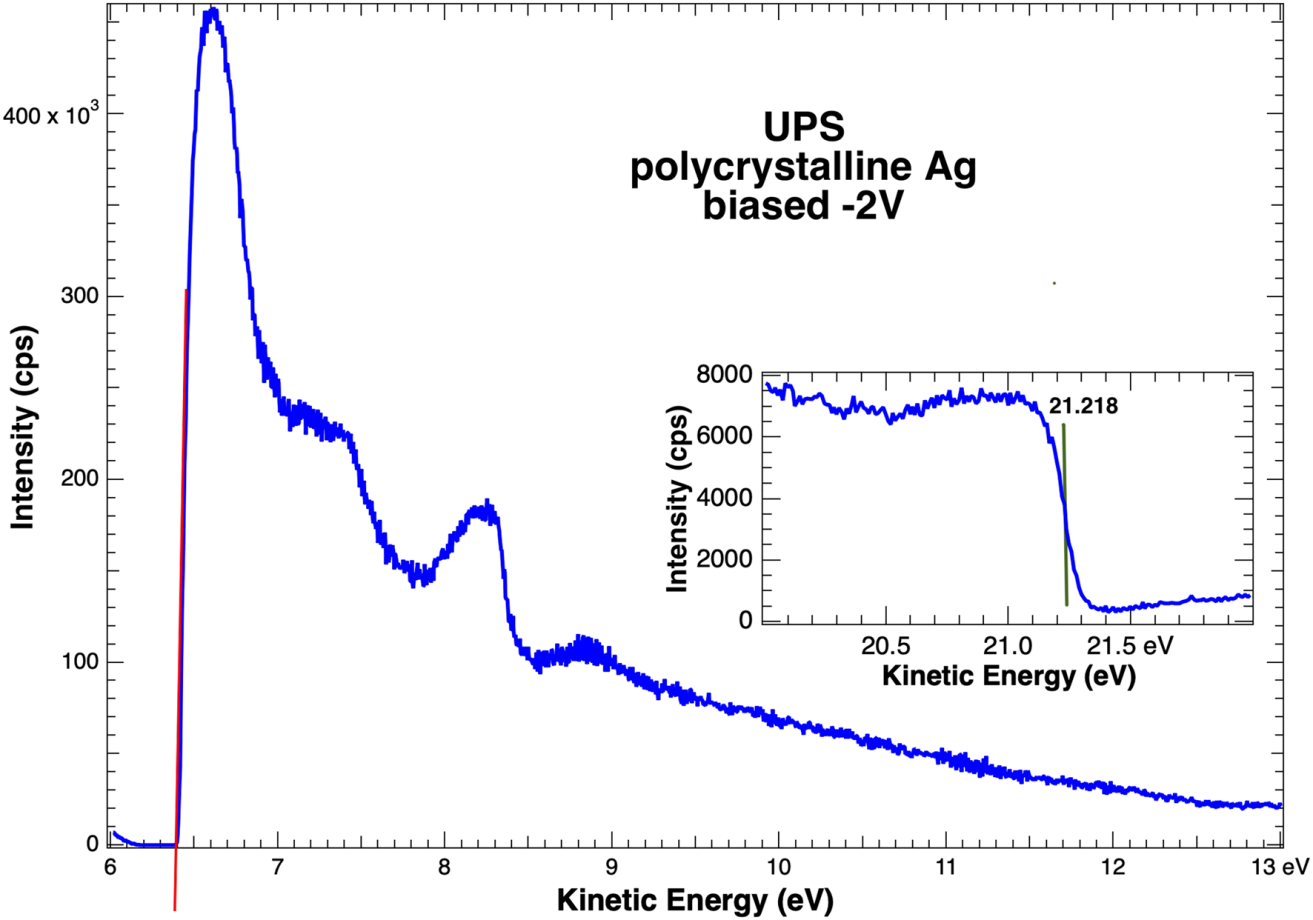
UPS onset spectrum from polycrystalline Ag with an intensity onset. The line is a fit with x-intercept at 6.40 eV. Subtracting 2 to account for the sample bias identifies the work function at 4.40 eV. The inset shows the Fermi Energy, defined to be 21.218 eV with the sample grounded.

A typical UPS onset spectrum for work function measurement is shown in Fig. 2. The sample is clean, polycrystalline Ag and the photon source is a He I discharge lamp with photon energy of 21.218 eV. The energy scale is initially set by grounding the sample and defining the kinetic energy of the Fermi edge electrons as the maximum available energy, 21.218 eV. For electrostatic energy analyzers, this is typically done by defining the work function of the analyzer with respect to ground. As noted above, the sample was biased by − 2.000 V which shifts the entire spectrum to higher kinetic energies to allow accurate and efficient electron detection at low kinetic energies.

The intensity onset, or SECO, is readily apparent at a kinetic energy slightly above 6 eV. A linear fit to the onset intersects the x-axis at 6.40 eV, which after accounting for the sample bias of − 2 provides the work function value of 4.40 eV. This measurement is in good agreement with published values^1^.

**Figure 3:**
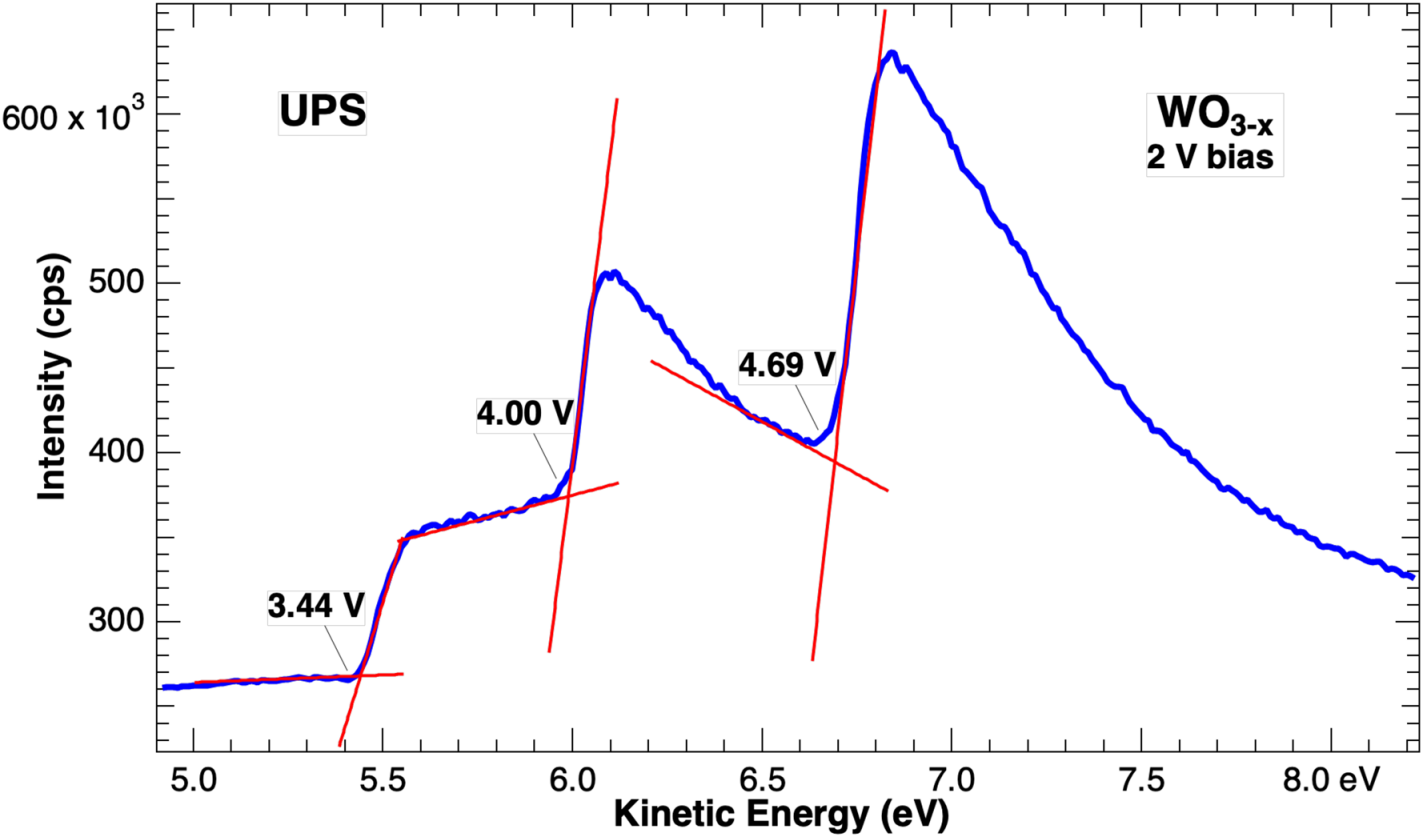
UPS intensity onset region from a tungsten oxide film with three steps each of which is a rapid intensity increase and could represent the material work function. Onsets are defined at the intersections of the linear fits and values are corrected for the − 2 V sample bias.

However, not all samples provide such a clear electron onset. Consider the spectrum provided in Fig. 3 from a WO_3−x_ film grown on an indium tin oxide covered glass slide. This material is of technological importance for photoelectro-catalytic conversion of methane and the work function is a key parameter in its role as a catalyst. In the spectrum, no single intensity onset is observed, instead three steps appear, each with the appearance of an onset. The figure includes lines fit at each step to identify the onset energy, with the near horizontal line representing a background from which the more vertical onset appears. The three onsets correspond to work functions of 3.44, 4.00, and 4.69 eV respectively. Previous studies found work functions for clean W ranging from 4.3–6 eV depending on crystal orientation^1^, and near stochiometric WO_3_ ranging from 5.3 to 6.3 eV^5^. The first two values from Fig. 3 are therefore unreasonably low.

**Figure 4:**
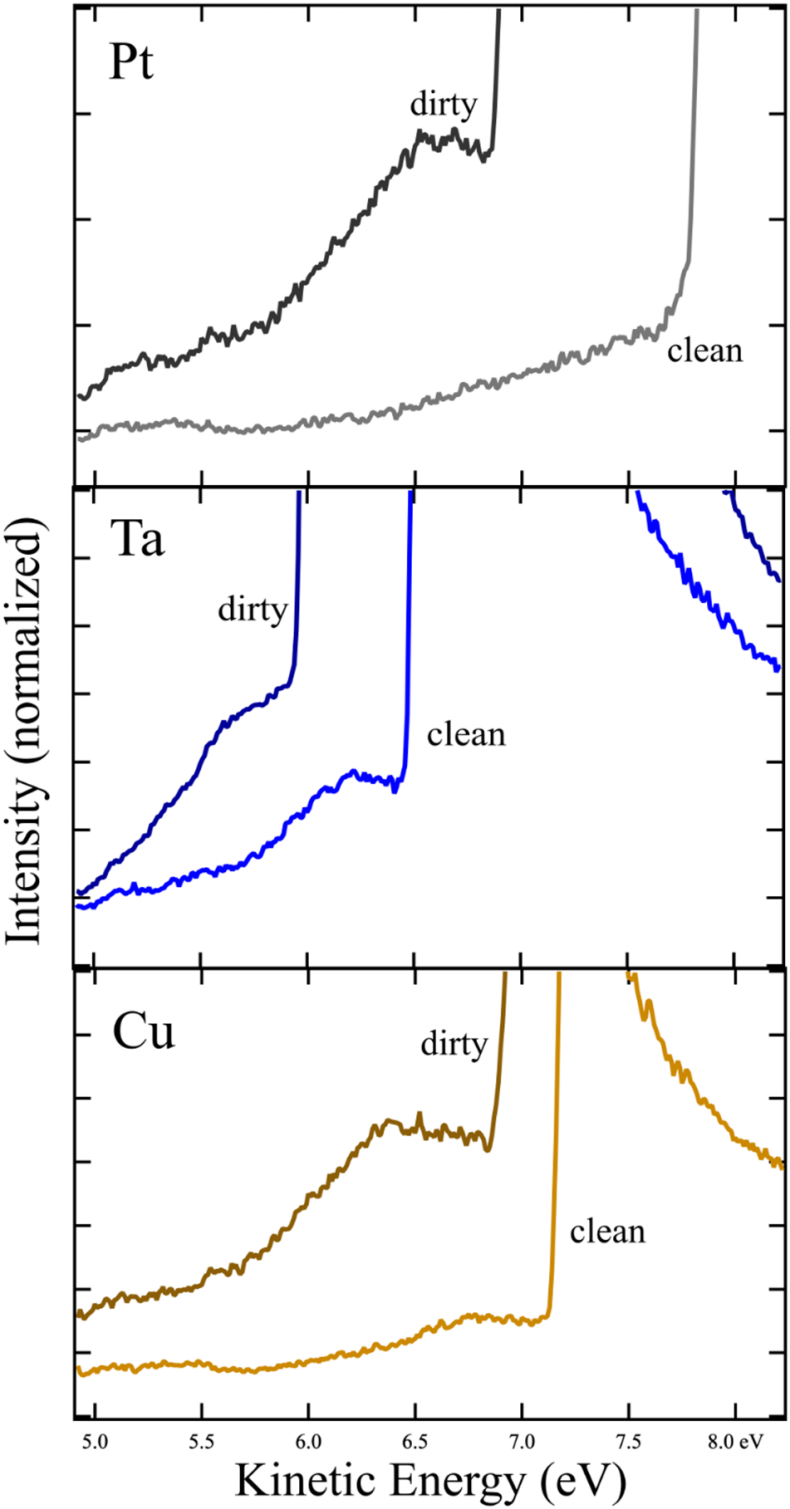
Onsets of UPS intensity (SECO) for clean and dirty transition metals, Pt, Ta, and Cu, where dirty means exposed to atmosphere. Samples were biased − 2 V. Dirty surfaces show additional intensity ~ 0.5 eV below the onset.

As the WO_3−x_ film had been exposed to air, UPS spectra from elemental metals with both clean and intentionally dirty surfaces were acquired and compared to explore the origins of intensities below the valid SECO. Here, “dirty” corresponds to clean samples exposed to atmosphere for a few minutes. Figure 4 shows results from polycrystalline Pt, Ta, and Cu surfaces. Again, samples have been biased − 2 V; work functions ascertained are 4.5–6 eV. Data intensities have been normalized to the maximum secondary intensity (not shown) for comparison. In these close-up views, some intensity is observed below the onset, more so for Ta than the others. However, as with the data for Ag in Fig. 1, clean surfaces show a single distinguishable onset in intensity with work function values comparable to those published.

Exposure to air does shift the intensity onset of the Pt, Ta, and Cu representing an expected modification of the work function. In addition to the shift, additional intensity is observed before the onset as seen in Fig. 4. In these cases, the onset is still easily identified; the additional intensity appears as a small peak at ~ 0.5 eV lower energy.

**Figure 5:**
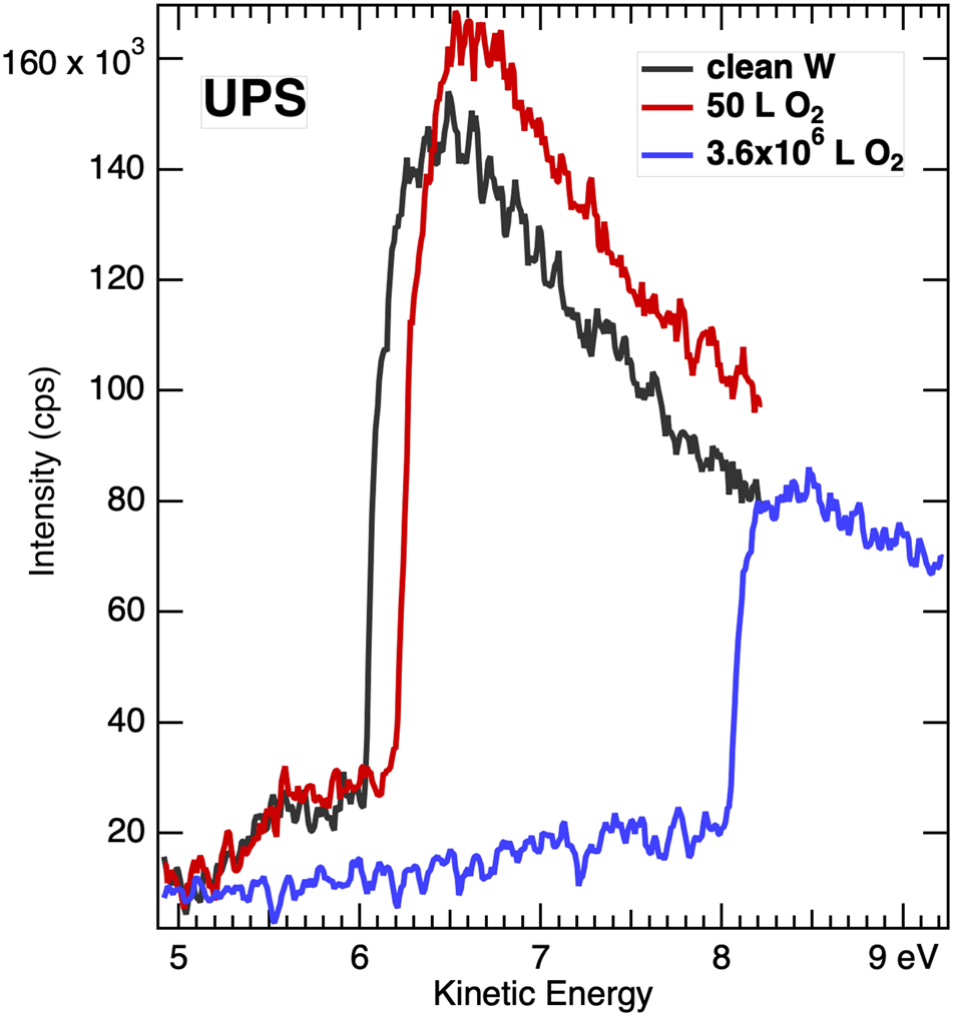
UPS onsets for clean and oxygen exposed polycrystalline W. From left to right the onsets are from clean, 50 L and 3.6 × 10^6^ L O_2_. A single onset is observed for all exposures. The sample is biased − 2 V.

To determine if the multiple onsets in Fig. 3 are specific to W or W with O, UPS onsets were measured for clean W and after exposures to pure O_2_. Results are shown in Fig. 5. A clear onset is seen for clean W although some intensity at lower kinetic energies is observed. Addition of 50 L and high exposure of 3.6 × 10^6^ L shifts the onset considerably, up to 2 eV for the higher exposure, but does not result in multiple onsets of intensity.

## Discussion

The UPS spectra reported here show that SECO onsets can be ambiguous, and that electron intensity can be observed at lower kinetic energies than the dominant onset. While the lower kinetic energy peaks in Pt, Ta, and Cu are relatively small, three onsets for WO_3−x_ films shown in Fig. 3 are comparable. Still since the simple picture of photoemission described in the introduction precludes any emission at energies below the work function onset, one might ask if these observations imply a lower work function for some parts of the sample or if the model is insufficient.

**Figure 6:**
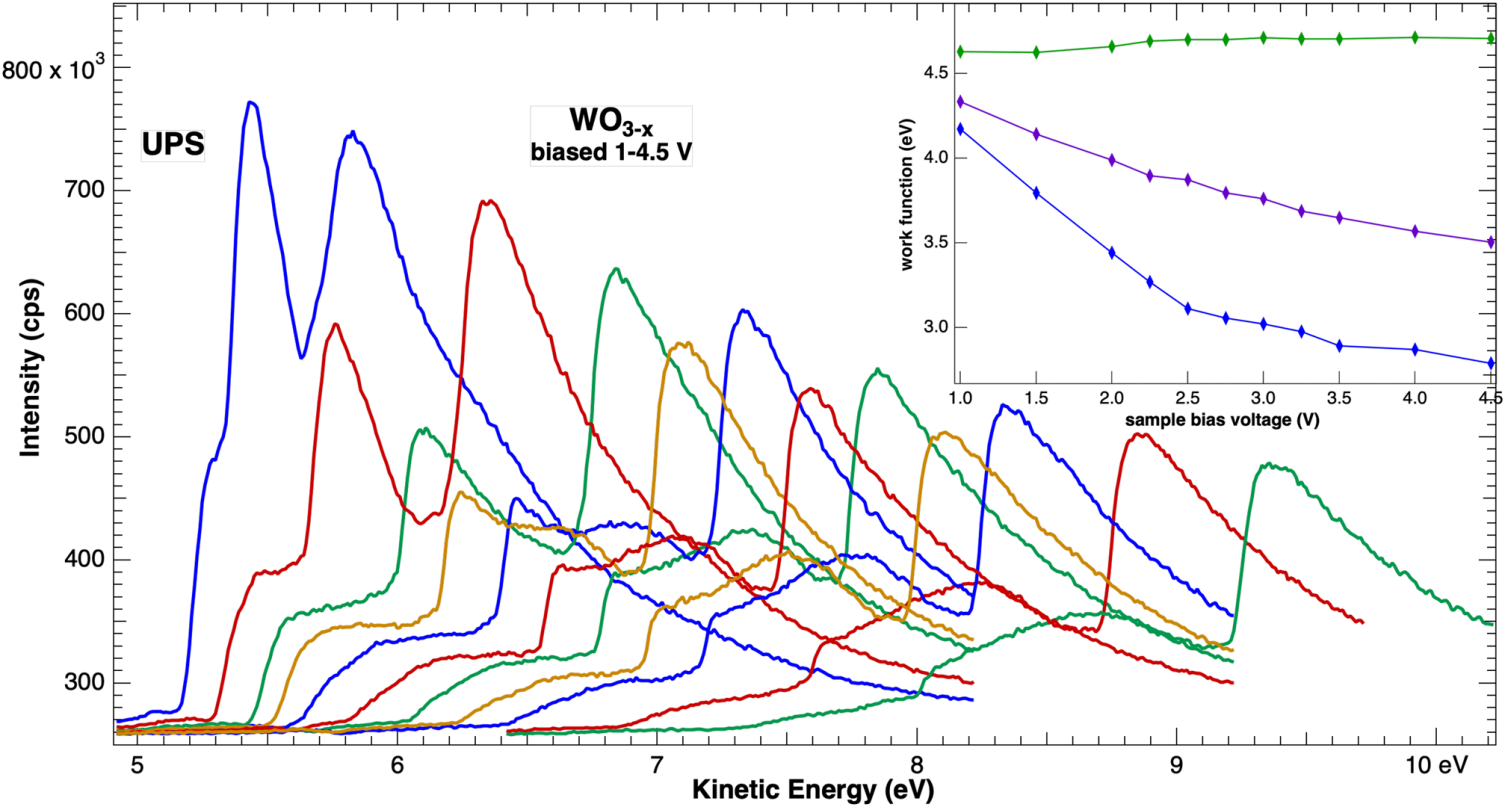
UPS onsets for WO_3−x_ films taken at different sample biases between − 1 and − 4.5 V (see text). Relative intensities and energies of the three onsets vary with bias. The inset plots the apparent work function (onset minus bias) derived at each bias voltage.

The first task is to identify proper onsets associated with the material work function. The most complex spectra to interpret is for WO_3−x_ films. As noted above, when compared with previously published values, work functions of 3.4 and 4.0 eV are unreasonably low. Insight into the onset correctly associated with the work function can be obtained by observing the spectra as a function of sample bias. For this system the results as shown in Fig. 6 for biases from left to right of − 1, − 1.5, − 2, − 2.25, − 2.5, − 2.75, − 3, − 3.25, − 3.5, − 4, and − 4.5 V. Closer steps were chosen in the range where experimental conditions were best, i.e. sufficient to accelerate the electrons to the detector, but not so high as to generate distorting electric fields. As distortions depend on the local electric fields this range will differ for each sample holder and electron analyzer lens geometry. The appropriate range can be determined by varying the bias in work function measurements on a clean metal, for example Ag. The lower bias limit is easily determined when the onset fully appears in the available scan range, while the upper limit can be identified when the onset becomes nonlinear with bias. In the experiments here, the working range was from about − 1 to − 5 V.

Intensity onsets in Fig. 6 evolve with the sample bias, specifically, the two lower energy steps diminish relative to the third as the bias increases. Furthermore, the spectra are not rigidly shifted by the bias and onsets do not align after correction for the bias value. This is illustrated in the inset to Fig. 6 where the derived work function obtained by subtracting the sample bias from the onset value is plotted against the bias. Only the highest onset provides a value, ~ 4.6 eV, that is independent of the bias. This supports the interpretation of the highest onset representing the true work function.

**Figure 7:**
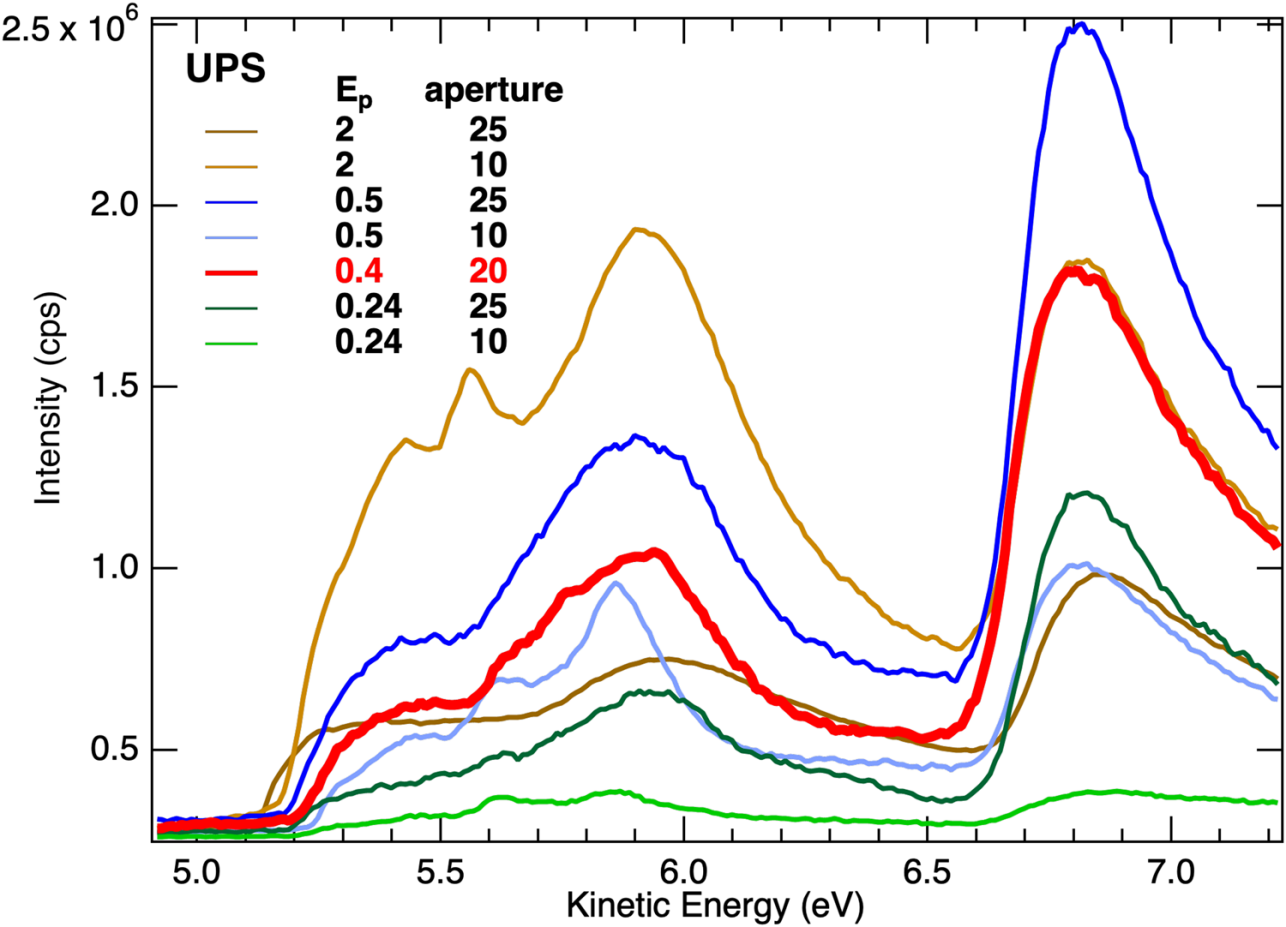
Optimization of hemispherical analyzer detector pass energy, E_p_ (eV), and acceptance aperture (mm) to minimize intensity below the onset at 6.6 eV. Smaller pass energies and apertures are effective, but minimizing both can reduce the total signal.

The second challenge is to identify experimental conditions that will emphasize the appropriate onset to determine the work function. Variation of the photon source, i.e. energy or intensity may be a benefit, however these parameters are not easily changed with conventional discharge lamps as employed in these experiments. In contrast, the electron detector, such as a hemispherical analyzer, typically offers control over pass energy, lens mode, detector voltage, aperture, and slit size. These were explored for the WO_3-x_ film with the goal of minimizing the intensities of the first two onsets relative to the third, while maintaining enough overall intensity to maintain an adequate signal-to-noise ratio. Of these parameters, the dominant impacts were found from pass energy and aperture settings. Figure 7 plots UPS spectra for some of these pass energy, E_p_, and aperture settings. By comparing spectra at the same E_p_ or aperture, the data show that reducing either limits the intensity below 6.5 eV relative to that above. At the same time, reducing both eliminates most of the total signal which can lead to signal-to-noise problems. For this experimental setup, an optimal balance was chosen at a pass energy of 0.4 eV and aperture of 20 mm, which is the bolder, red line in the figure. This choice maximizes the relative intensity in the higher energy peak without sacrificing too much signal. It is recommended that the minimum pass energy and aperture settings be employed that provide an acceptable signal-to-noise, which will depend on the source intensity and measurement time available.

Finally, a discussion of the origins of electrons at kinetic energies below the onset (SECO) is helpful for future measurements. Examples of multiple SECOs have been previously reported for Au after exposure to air and partial sputtering^4^, for an ITO mesh on Au substrate^6^, for partially annealed ZnO films^7^ and for water absorption on defect modified TiO_2_^8^. In these cases, additional onsets were attributed to heterogeneous surfaces where second onsets represented correct work functions of different material components. As the photoemission process is a local phenomenon, surfaces with two or more materials exposed within the spot size of the excitation will naturally exhibit multiple onsets representing the work functions of the components. For WO_3−x_, however, the additional onsets are at too low a kinetic energy to reasonably attribute them to a work function for this system, where values range from 4.3 eV for W^1^ to 6.3 eV for WO_3_^5^. This makes inhomogeneity an unlikely explanation for the multiple onsets here.

A different origin of multiple SECOs has been proposed in which photoemitted electrons lose energy to surface excitations while exiting the material. This lowers their kinetic energy, so they appear below the SECO in UPS spectra. This model has been used to explain results observed for carbon nanospikes coated with hydrocarbons^9^. In that system UPS spectra showed a peak below the onset which was not attributed to inhomogeneity nor considered representative of a true work function SECO. The peak disappeared after annealing and returned with addition of hydrocarbons. The peak was located ~ 0.4 eV below the SECO, an energy typical for excitation of a dipole-active C–H vibrational stretching mode, offering a plausible avenue for energy loss by exciting electrons. This mechanism appears appropriate for work function spectra of the elemental metal surfaces of Pt, Ta and Cu after exposure to air as these surfaces are saturated with absorbates with low energy excitations. WO_3-x_ has a strong surface plasmon resonance near 0.8 eV, Ref.^10^ which is close to the energy spacing of the multiple onsets in Fig. 3. As a dipole excitation, a plasmon has a large electronic cross-section consistent with the intensity of the features seen here. However, an energy loss model is probably inadequate for this system. Kinetic energies of 0.8 eV and greater are not available to electrons minimally emitted from the surface making excitations of this magnitude inaccessible before the electrons are too far from the surface to interact.

A third mechanism that can produce intensity below the SECO is surface charging. On a non-conducting or ungrounded material, photoemission removes electrons leaving a positively charged surface. This increases the local bias (reducing the magnitude of the negative bias applied) and shifts spectral intensity to lower values. This is an unexpected mechanism for WO_3-x_ films; the resistivity of WO_3−x_ is a few mΩ-cm^11^ and macroscopic measurements from sample to ground were under 5 ohms. Spectra were reproducible both from the same sample and different films. Nevertheless, local areas of the film could be poorly grounded, and this mechanism may better explain the observations than the previous two. The somewhat discrete peak spacing of ~ 0.7 eV remains to be explained.

Either energy loss to excitations or surface charging could explain the observed bias dependence for peaks below the correct onset. As noted above, axial sample-analyzer symmetry is important particularly at higher sample biases where the electric fields are greater and nonuniform off axis. Energy loss and charging processes would be more influenced by off-axis fields since energy reductions make electrons slower and more susceptible to electric fields.

In summary, work function measurements made using UPS can provide absolute values, however, some samples can produce spectra that are difficult to interpret due to additional intensity below the true secondary electron cutoff. In general, disordered absorbates add intensity below the cutoff for the transition metal surfaces studied, and much stronger intensities were observed for WO_3-x_ films. This lower kinetic energy intensity can occur due to energy losses after photoelectrons are generated or sample charging. Invalid onsets can be minimized by optimizing detection parameters, for example limiting analyzer acceptance angles and pass energy. True work functions can be determined by examining the onsets as the sample bias is varied.

## Methods

Bulk samples were polycrystalline metals of high purity (W, Ta, and Pt: 99.95%; Cu: 99.999%) about 1 cm in width and polished to a mirror finish. WO_3−y_ thin films were grown on ITO coated glass by Ho Kun Woo and Lili Cai at the University of Illinois, Urbana-Champaign using flame vapor deposition^12,13^. Scanning electron microscopy, transmission electron microscopy and x-ray diffraction analyses reveal the films are composed of a dense array of 1D vertically aligned nanotubes with monoclinic WO_3_ structure. XPS measurements found no hydroxyl peak (~ 532 eV), i.e. no H_x_WO_3_ phase was formed during growth^14^. Films were supported and electrically connected to the sample plate by tantalum wires at four corners. Resistivity across the film and to ground was less than 5 ohms as determined by a Fluke 179 multimeter.

UPS measurements were made in the Oak Ridge National Laboratory NanoTransport ultrahigh vacuum chamber^15^ with an operating pressure of 2 × 10^−10^ torr. Ultraviolet light for UPS was generated with a SPECS UVS300 lamp operated at 5 × 10^−5^ Torr and 1 A emission to maximize He I radiation at 21.218 eV. The He I:He II intensity ratio was about 5000:1. Electron detection was performed with a SPECS Phoibos 150 hemispherical analyzer operated at 0.4 eV pass energy. The sample normal was oriented toward the electron detector.

Work functions were determined by identifying the secondary electron cut-off (SECO) energy in UPS. This is identical to measuring the photoemission total band width with the sample grounded and Fermi energy fixed to 21.218 eV kinetic energy (0 eV binding energy). The Fermi energy position was calibrated with a sputtered and annealed Ag sample. In practice a proper secondary cut-off energy can only be measured by negatively biasing the sample, since the electrostatic electron detector cannot detect near 0 eV electrons. In these experiments, samples were biased between 0 and 4.5 V in 0.5 V increments to avoid systematic detector error. Measurements are reported for − 2 V biases where the SECO shifts were linear. Following common practice, the SECO was determined by the intersection of two linear fits to the data reflecting the background and the onset.

## Data Availability

The Department of Energy will provide public access to these results of federally sponsored research in accordance with the DOE Public Access Plan (http://energy.gov/downloads/doe-public-access-plan).
